# Experience that Much Work Produces Many Reinforcers Makes the Sunk Cost Fallacy in Pigeons: A Preliminary Test

**DOI:** 10.3389/fpsyg.2016.00363

**Published:** 2016-03-16

**Authors:** Shun Fujimaki, Takayuki Sakagami

**Affiliations:** ^1^Department of Psychology, Keio UniversityTokyo, Japan; ^2^Japan Society for the Promotion of ScienceTokyo, Japan

**Keywords:** sunk cost, Concorde fallacy, operant conditioning, suboptimal choice, behavioral history, pigeons

## Abstract

The sunk cost fallacy is one of the irrational choice behaviors robustly observed in humans. This fallacy can be defined as a preference for a higher-cost alternative to a lower-cost one after previous investment in a higher-cost alternative. The present study examined this irrational choice by exposing pigeons to several types of trials with differently illuminated colors. We prepared three types of non-choice trials for experiencing different outcomes after presenting same or different colors as alternatives and three types of choice trials for testing whether pigeons demonstrated irrational choice. In non-choice trials, animals experienced either of the following: (1) no reinforcement after the presentation of an unrelated colored stimulus to the alternatives used in the choice situation, (2) no reinforcement after investment in the lower-cost alternative, or (3) reinforcement or no reinforcement after investment in the higher-cost alternative. In choice trials, animals were required to choose in the following three situations: (A) higher-cost vs. lower-cost alternatives, (B) higher-cost vs. lower-cost ones after some investment in the higher-cost alternative, and (C) higher-cost vs. lower-cost alternatives after the presentation of an unrelated colored stimulus. From the definition of the sunk cost fallacy, we assumed that animals would exhibit this fallacy if they preferred the higher-cost alternative in situation (B) compared with (A) or (C). We made several conditions, each of which comprised various combinations of three types of non-choice trials and tested their preference in three choice trials. Pigeons committed the sunk cost fallacy only in the condition that contained non-choice trials (3), i.e., pigeons experienced reinforcement after investing in the higher-cost alternative. This result suggests that sunk cost fallacy might be caused by the experiences of reinforcement after investing in the higher-cost alternative.

## Introduction

The sunk cost fallacy is defined as a tendency to continue an endeavor once an investment in money, effort, or time has been made (Arkes and Blumer, 1985). This effect is considered as an irrational choice behavior from the perspective of classical economic and normative decision theories (Garland and Newport, 1991). Many studies have been conducted on humans (e.g., Arkes and Blumer, 1985; Staw and Hoang, 1995; Arkes, 1996; Cunha and Caldieraro, 2009) and non- human animals (e.g., Dawkins and Brockmann, 1980; Lavery, 1995; Navarro and Fantino, 2005; de la Piedad et al., 2006; Watanabe, 2009; Magalhães and White, 2014).

In human studies, Arkes and Blumer (1985) systematically examined the sunk cost fallacy using the following types of scenarios.

As the president of an airline company, you have invested 10 million dollars of the company’s money into a research project. The purpose was to build a plane that would not be detected by conventional radar, in other words, a radar-blank plane. When the project is 90% completed, another firm begins marketing a plane that cannot be detected by radar. Also, it is apparent that their plane is much faster and far more economical than the plane your company is building. The question is: should you invest the last 10% of the research funds to finish your radar-blank plane?

In this situation, quitting the investment is a rational decision, because it is apparent that the other firm’s plane is much faster and far more economical. Nevertheless, in Arkes and Blumer’s (1985) studies, many participants decided to continue the investment. In other words, they committed the sunk cost fallacy. However, majority of the participants chose not to invest in the airplane’s construction in the similar questionnaire, where nothing had been initially invested. These results suggest that 90% of the initial investment (i.e., the sunk cost) was the cause of continuing the investment.

In non-human animals, the sunk cost fallacy has been called the *Concorde fallacy* and studied in behavioral ecology and evolutionary biology. Several early studies showed evidence that animals living in the wild commit the Concorde fallacy (e.g., Weatherhead, 1979; Dawkins and Brockmann, 1980; Lavery, 1995). However, Arkes and Ayton (1999) indicated that any case of those findings could be explained from the perspective of future cost and benefit, and not only past investment. They eventually concluded that there is no clear evidence for the tendency to commit the Concorde fallacy in non-human animals.

Contrary to this, more recent laboratory studies have tried to find evidence of the sunk cost fallacy in situations that exclude the explanations of future cost and benefit (e.g., Navarro and Fantino, 2005; de la Piedad et al., 2006; Pattison et al., 2012; Magalhães and White, 2013, 2014). Navarro and Fantino (2005) first reported the sunk cost fallacy using pigeons in a laboratory study. They used two response keys—a food key and an escape key. During each trial, one of the four fixed-ratio (FR) schedules was randomly assigned to the food key according to their probability (FR 10 on 50%, FR 40 on 25%, FR 80 on 12.5%, and FR 160 on 12.5%). Responses to the food key produced the food after the completion of the assigned FR value, which was then followed by a new trial. A response to the escape key immediately terminated the current trial and was followed by a new trial. In both cases, the FR value of the food key was reselected at the start of the trial according to each probability. The optimal and suboptimal choice was defined on the basis of an expected value of the food key. At the start of the trial, the expected value was 45 (i.e., 0.5 × 10 + 0.25 × 40 + 0.125 × 80 + 0.125 × 160). If no food was presented after 10 responses, the expected value became 70 (0.5 × 30 + 0.25 × 70 + 0.25 × 150), and after 40 responses, it became 80 (0.5 × 40 + 0.5 × 120). The optimal strategy was therefore defined as choosing the escape key if no food was presented after 10 responses. The suboptimal choice (i.e., the sunk cost fallacy) was defined as getting food other than the minimum FR value.

Navarro and Fantino (2005) conducted this procedure with the stimulus change present condition and the stimulus change absent condition. At the start of the trial, the food key color turned white in both conditions. In the stimulus change present condition, the color turned red, blue, and green just after each 10, 40, 80 responses, and the color was constant throughout the sessions in the stimulus change absent condition. When stimulus changes were present, all pigeons chose the escape key after 10 responses. On the contrary, when these stimulus changes were absent, three out of four pigeons chose the food key even after 10 responses. Many following studies using a similar procedure succeeded in replicating Navarro and Fantino’s (2005) results (e.g., Avila-Santibañez et al., 2010; Macaskill and Hackenberg, 2012a,b; Magalhães et al., 2012).

However, the sunk cost fallacy demonstrated by these studies seems inconsistent with the sunk cost fallacy in human studies. The main difference is whether exists or not the signal that indicates continuing the investment is irrational. For example, in Arkes and Blumer’s (1985) questionnaire, the existence of a much faster and more economical plane signals that continuing the investment is irrational. Although this situation corresponds to the stimulus change condition of Navarro and Fantino’s (2005) procedure, no pigeons committed the sunk cost fallacy under this condition (see also Macaskill and Hackenberg, 2012b, Experiment 3). Instead, they regarded the persistence in the food key under the stimulus change absent condition as the sunk cost fallacy. This point is a crucial difference between human and animal studies conducted in context of operant conditioning.

This discrepancy might stem from the somewhat unclear definition of the sunk cost fallacy. Since the sunk cost fallacy might occur in various situations, it is difficult to determine a uniform definition. However, if we consider the sunk cost fallacy as an *irrational behavior*, the irrationality could be characterized by at least three requirements. First, continuing the investment because of the past investment (i.e., the sunk cost), not because of future cost and benefit. Second, continuing the investment results in a failure of profit maximization or loss minimization. Third, the existence of a signal that indicates continuing the investment is irrational.

All studies followed by Navarro and Fantino’s (2005) procedure did not satisfy the third requirement. In the stimulus change absent condition, it is unclear whether the subject discriminates that persisting in the current choice is rational or irrational. Contrary to this, in the instance of constructing the supersonic airplane *Concorde*, for example, the British and French governments continued their investment even after it was apparent that the project was unprofitable. This has sometimes been used as a typical instance of the sunk cost fallacy (e.g., Dawkins and Carlisle, 1976). However, if the governments had not known that continuing the investment would fail; whether their decision would be considered an example of the irrational decision-making? Perhaps, the answer is no. Their decision to continue the investment was considered irrational because both governments knew that it was unprofitable. Had they not known this fact, their decision might have not been considered irrational. Altogether, the phenomenon referred to as the sunk cost fallacy is not always consistent across animal and human studies. This discrepancy makes it difficult to explore the common mechanism across species underlying the sunk cost fallacy.

To overcome this problem, the present study examined the sunk cost fallacy using pigeons under experimental conditions that met all three requirements mentioned above. We also attempted to examine the independent variable causing the sunk cost fallacy across species. A problem of what could be a common independent variable has not been well examined. Given that the sunk cost fallacy is a phenomenon affected by past investment, behavioral experiences or behavioral histories could be the candidate. Macaskill and Hackenberg (2012a) reported that pigeons were less likely to commit the sunk cost fallacy when they had a history of choosing escape key results in a reinforcement by the shortest number of responses (also see Navarro and Fantino, 2005, Experiment 5; Macaskill and Hackenberg, 2012b; Avila et al., 2013). If certain behavioral experiences could influence prompting optimal choice behavior, there would also be specific behavioral experiences causing the sunk cost fallacy. In human studies, Goltz (1992, 1993) have shown that such histories exist. However, few studies examined this possibility in animal studies, while having indicated that doing so is important (e.g., Avila et al., 2013). We therefore examined whether particular behavioral experiences would cause the sunk cost fallacy.

## Experiment 1

We prepared three types of non-choice trials for experiencing different outcomes after presenting the same or different colors as lower- or higher-cost alternatives. We also prepared three types of probes for testing whether the subjects demonstrated irrational choices. In non-choice trials, animals experienced the following situations: (1) no reinforcement after the presentation of an unrelated color stimulus to the alternatives used in choice situations, (2) no reinforcement after the responses to the lower-cost alternative, and (3) reinforcement or no reinforcement after the responses to the higher-cost alternative. In probes, they were required to choose one of the alternatives in the following three situations. In probe A, lower-cost colored vs. higher-cost colored alternatives; in probe B, lower-cost vs. higher-cost alternatives after some responses to the higher-cost alternatives; in probe C, lower-cost vs. higher-cost alternatives after the presentation of an unrelated colored stimulus. From the definition of the sunk cost fallacy suggested above, we considered that the sunk cost fallacy occurred when pigeons preferred the higher-cost alternative only in probe B and not in probe A and C.

### Methods

#### Subjects

The subjects were four pigeons (*Columba livia*) maintained at about 80% of their free-feeding weights. They were housed individually with a 12:12 h light/dark cycle (lights on 08:00 a.m.) and had free access to water and grit in the home cage. All subjects had previous experiences with various experimental procedures. This research was approved by Laboratory Animal Center, Keio University School of Medicine and conducted following their guidelines.

#### Apparatus

Four operant chambers, 32 cm long, 25 cm wide, and 33 cm high were used. Each chamber was housed in a sound-attenuating box with a ventilation fan. During experimental sessions, white noise presented in the box masked extraneous noise and a house light on the rear wall 30 cm above the grid floor provided general illumination. Each chamber had three response keys on the front wall 26 cm above the grid floor. Each key was 3 cm in diameter and 6 cm apart from each other (center to center) and could be illuminated with lights of different colors. Food reinforcement was access to mixed grain delivered by a food hopper located behind a 6 cm square aperture centered on the front panel 5 cm above the floor. During reinforcement, the aperture was illuminated white and the houselight and all keylights were turned off. Event scheduling and data recording were controlled by a computer using Visual Basic 2005 Express Edition software.

### Procedure

#### Preliminary Training

The subjects were initially trained to key pecking responses on an FR schedule. The response requirement was gradually increased from 1 to 20 times across sessions. Sessions continued until 60 reinforcers were delivered. This was conducted 6 days a week at approximately the same time each day.

It was necessary to determine the value of the lower- and higher-cost alternatives before the main training. We defined the lower-cost value as FR 2 and defined the higher-cost value as the point at which the pigeons chose the lower-cost key more than 90% in the choice situation where both alternatives were simultaneously available. It was quite possible that the higher-cost value meeting this definition was different for each pigeon. We therefore determined it for each subject using the adjusting procedure (Mazur, 1987).

Each session of the adjusting procedure lasted 96 trials, and the trials were divided into 24 blocks of four trials. The first two trials of each block were forced-choice trials and the last two trials were free-choice trials. In forced-choice trials, one of the side keys was illuminated with either the lower- or higher-cost color. The presentation order and the location of these colors were randomly assigned across blocks. In free-choice trials, both side keys were simultaneously illuminated with either the lower- or higher-cost color. The location of the colors was quasi-randomly assigned for each free-choice trial. The first peck on either side key served as a choice response and extinguished the other side key. Completion of either key’s requirement provided 3-s access to food. The completion of the second free-choice trial was followed by a new block after a 60-s interval.

At the start of each session, the value of the lower- and higher-cost key was FR 2 and FR 15, respectively. The value of the lower-cost key was constant throughout the sessions, but the value of the higher-cost key changed depending on the choice in the free-choice trial. If the subject chose the higher-cost key for both free-choice trials, its value was increased by 1 for the next block (i.e., FR 15 + 1 = FR 16). If the subject chose the lower-cost key on both free-choice trials, the value of the higher-cost key was decreased by 1 (FR 15 - 1 = FR 14). If the subject chose each key on one trial, the value was the same as that of the previous block (FR 15). The minimum value of the higher-cost was FR 3 and there was no limitation on the maximum value.

We calculated the percentage choice of the higher-cost key at each higher-cost value using data during the last five sessions. The percentage choice was calculated by dividing the number of choices of the higher-cost key by the total number of choices at each higher-cost value. For example, if the number of choices for the higher-cost key was five, and the total number of choices was 20 when the higher-cost value was FR 10, the percentage choice for the higher-cost key was 25%. The condition lasted for a minimum of 10 sessions and terminated when there was at least one higher-cost value where the percentage choice of the higher-cost key was less than 10%. If there were several candidate values, the higher-cost was defined as the minimum value of those. As a result, the value of the higher-cost was determined FR 13, FR 4, FR 5, and FR 9 for subject B12, B14, C12, and H21, respectively.

#### Main Training

The experiment included six conditions. Each condition comprised the combination of non-choice trials and probe trials. The experimental session ended after 112 trials across conditions. Non-choice trials and probe trials were quasi-randomly presented within a session, and a 15-s inter-trial interval (ITI) preceded the next trial.

**FIGURE 1:**
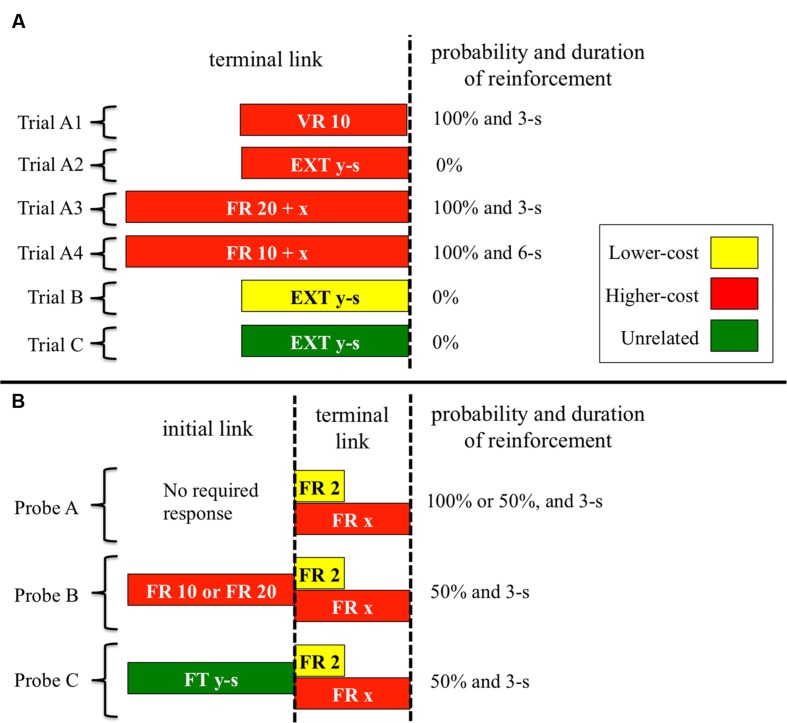
**Schematic of non-choice trials **(A)** and probe trials **(B)** used across Experiments.** The acronym “EXT” represents the extinction schedule, and the “*x*” and “*y*” represent a value of FR schedule and the duration of the extinction and FT schedules, respectively. Trial A1 and A2 were used in Experiment 1, trial A3 was used in Experiment 2, and trial A4 was used in Experiment 3. The reinforcement probability of probe A was 100% in Experiment 1 and 50% in Experiments 2 and 3. The initial link of probe B was FR 20 in Experiments 1 and 2, and FR 10 in Experiment 3. The durations of trials B and C, and the initial link of probe C were different for each subject. See text for further details.

Non-choice trials were of the following four types (see **Figure 1A**). In trial A1, the center key was illuminated with red (same color as the higher-cost key) and the response was reinforced on variable-ratio (VR) 10 schedule. The response requirement for reinforcement ranged between 5 and 15 responses in each trial. Even after reinforcement, trial A1 continued until the 20th response. For example, if food was presented at the 10th response, the trial was terminated after additional 10 responses. In trials A2, B, and C, the center key was illuminated with red, yellow (same color as the lower-cost key), and green (unrelated to both the lower- and higher-cost keys), respectively. The extinction schedule was effective in these trials and the duration was determined for each subject by calculating the mean completion time of FR 20 within the 20th and 80th percentile over the last five sessions in pre-training. The durations were 8.2, 13.4, 13.6, and 9.4-s in B12, B14, C12, and H21, respectively.

Probe trials were of the following three types (see **Figure 1B**). In Probe A, pigeons were exposed to a choice situation between the lower- and higher-cost keys. At the start of the trial, yellow and red were presented on either the left or right side key. The first peck on either side key served as a choice response and extinguished the other side key. The reinforcer was always presented for 3-s when pigeons met the response requirement. In probe B, the higher-cost color was presented on the center key in the initial link. After 20 responses, the lower- and higher-cost colors were presented on either side key in the same manner as probe A. In probe C, green was presented on the center key in the initial link. The initial link was the same as that of trial C, so that after the specific period ended (i.e., fixed time, FT, schedule), pigeons were exposed to the choice situation identical to probe A. The terminal link of probes B and C was the same as probe A, except for the reinforcement probability—the probability was 50% in both probe B and probe C. In all probes, the location of the colors in the choice situation was randomly assigned for every trial.

All pigeons were exposed to the following six conditions (see **Table 1**). Each condition lasted for a minimum of 10 sessions and until no systematic trend in preference was observed for at least three consecutive sessions.

**Table 1:** The number of each non-choice trial and probe trial in each condition of Experiments 1–3.

Trial Types	Experiment 1						Experiment 2				Experiment 3			
			
	I	II	III	IV	V	VI	I	II	III	IV	I	II	III	IV
Trial A1	0	32	0	32	0	0	0	0	0	0	0	0	0	0
Trial A2	0	0	16	0	0	0	0	0	0	0	0	0	0	0
Trial A3	0	0	0	0	0	0	0	0	48	24	0	0	0	0
Trial A4	0	0	0	0	0	0	0	0	0	0	0	0	48	24
Trial B	0	0	0	32	16	32	0	48	0	24	0	48	0	24
Trial C	32	32	16	0	16	0	64	16	16	16	64	16	16	16
Probe A	64	32	64	32	64	64	8	8	8	8	8	8	8	8
Probe B	8	8	8	8	8	8	8	8	8	8	8	8	8	8
Probe C	8	8	8	8	8	8	8	8	8	8	8	8	8	8


*Each trial type was presented in a quasi-random order for each session. Daily sessions ended after 112 trials in Experiment 1 and 88 trials in Experiments 2 and 3. The reinforcement probability of probe A was 100% in Experiment 1, and 50% in Experiments 2 and 3.*

#### Condition I (Baseline Condition)

Condition I consisted of trial C and three types of probes. In this condition, pigeons did not have any behavioral experiences related to the lower- and higher-cost keys. The purpose of condition I was twofold. First, we tested the value of the higher cost, which was determined using the adjusting procedure. The criterion of the appropriate value was that the percentage choice of the higher-cost key in probe A was stable at less than 10%. If the percentage choice was stable at more than 10%, the higher-cost value was gradually increased until it was stable at less than 10%. The second purpose was to examine whether the pigeons committed the sunk cost fallacy without any behavioral experiences.

#### Condition II

Condition II consisted of trials A1 and C, and probes. The purpose of condition II was to examine whether the experiences created by trial A1 succeeded in causing the sunk cost fallacy. In trial A1, responses to the higher-cost key were reinforced on VR 10. This experience was expected to increase the preference for the higher-cost key (see, e.g., Fantino, 1967; Pattison et al., 2012).

#### Condition III

Condition III consisted of trials A2 and C, and probes. The purpose of condition III was to test whether the pigeons’ preference decreased to less than the previous condition’s result by the experience of trial A2. In trial A2, all responses to the higher-cost key were extinguished; this was expected to have the effect of devaluating the higher-cost key.

#### Condition IV

Condition IV consisted of trials A1 and B, and probes. Since this condition contained trials A1 and B, pigeons had two types of experiences—responses to the higher-cost color produced reinforcers (trial A1) and responses to the lower-cost color were extinguished (trial B). We examined whether the combination of two types of experiences caused the sunk cost fallacy.

#### Conditions V and VI

Conditions V and VI consisted of trial B and probes. In addition, trial C was included only in Condition VI. The main difference between these conditions was the number of trial B so that we examined the effect of experience created by trial B and the number of presentation of trial B on the higher-cost preference.

It should be noted that the number of probe A in conditions II and IV differed from the others in order to preclude confounding by differences in obtained reinforcement: reinforcer was delivered on 100% in trial A1 so that if the number of trial A1 had been manipulated while holding the number of probe A constant, the effect of introducing trial A1 would have been confounded by the differences in obtained reinforcement. Changing one variable at a time across conditions is widely known as a cardinal rule of single-subject research (Barlow et al., 2009). We therefore decreased the number of probe A in conditions II and IV to equate the obtained reinforcement across conditions.

### Results and Discussion

**FIGURE 2:**
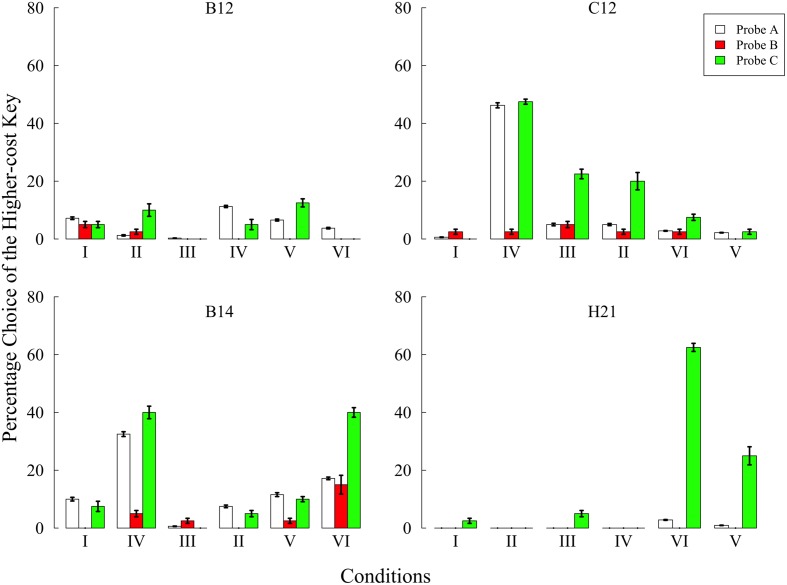
**Preference for the higher-cost key in each probe over the last five sessions of each condition in Experiment 1.** Choice percentages were calculated in each probe by dividing the number of choices of the higher-cost key by the total number of choices. Error bars represent standard errors.

**Figure 2** shows the preference for the higher-cost key in each condition. In condition I, B14 and C12 preferred the higher-cost key more than 10% of all choice opportunities in probe A. We therefore gradually increased the higher-cost value and changed from FR 4 to FR 14 in B14 and from FR 5 to FR 8 in C12, respectively. Eventually, no pigeons showed a preference for the higher-cost key in probe A. In addition, all pigeons did not prefer the higher-cost key in the other probes. These results show that pigeons do not commit the sunk cost fallacy when they have no experiences related to the lower- and/or higher-cost keys.

In the other conditions, some pigeons showed a preference for the higher-cost key. In condition IV, B14 and C12 showed a remarkable increase of preference in probes A and C. The preferences in these pigeons decreased in condition III, which was followed by condition IV. Thus, the increased preference was caused by the effect of behavioral experiences created in condition IV. In another case, B14 and H21 also showed the higher-cost preference in condition VI and only H21 showed similar results in condition V. However, these increased preferences were observed only in probe A or probe C, not in probe B. During condition VI, only B14 increased the higher-cost preference in probe B compared with other conditions, but the preference was lower than that of probes A and C. Taken together, the present experiment failed to demonstrate the sunk cost fallacy under any condition while showing, nonetheless, that behavioral experiences could change the subjects’ preference.

In the present study, it is highly possible that only trial B had the effect of increasing the higher-cost preference. This possibility was supported by the following results; the increased preference in probes A and C was observed in the conditions, including trial B. Moreover, no pigeons preferred the higher-cost key in any probes in condition II including trial A1. These results supported the view that the increased preference observed in condition IV was caused only by trial B. Trial A1 had no influence in increasing the preference. Trial A1 might have created some aversion to the higher-cost key rather than increasing preference. As noted above, trial A1 continued until the 20th response even after reinforcement. It is possible that the required responses after the reinforcement were aversive and that this aversion prevented the increase in the higher-cost preference.

We further examined the position preference using a two-tailed binomial test. **Table 2** shows the mean percentage choice of the left key in each probe over the last five sessions. C12 preferred the left key in probe A and preferred the right key in probe C of Condition IV, and H21 preferred the left key in probe C of Condition V and VI. Those performances were significantly above chance level (*p*s < 0.01). Furthermore, the position preference was related to the higher-cost preference. In H21’s case, preference for the left key was increased in probe C in conditions V and VI, and the percentage choice of the higher-cost key was thereby increased. It is noteworthy that this type of position preference occurred only in the condition including trial B. Although the cause of the position preference was unclear, this anomaly might reflect the decreased preference for the lower-cost key.

**Table 2:** The order of the condition, the number of sessions, and the mean percentage choice of the left key in each probe of each condition in Experiment 1.

Subjects	Conditions	Number of sessions	Percentage choice of the left key		
			
			Probe A	Probe B	Probe C
B12	I	16	0.46 (0.05)	0.45 (0.19)	0.40 (0.10)
	II	13	0.48 (0.06)	0.43 (0.23)	0.48 (0.19)
	III	12	0.51 (0.01)	0.50 (0.09)	0.43 (0.23)
	IV	20	0.56 (0.06)	0.48 (0.01)	0.40 (0.21)
	V	15	0.58 (0.08)^∗∗^	0.53 (0.14)	0.53 (0.16)
	VI	16	0.46 (0.03)	0.43 (0.14)	0.43 (0.21)
B14	I	22	0.56 (0.13)^∗^	0.43 (0.21)	0.43 (0.14)
	IV	14	0.70 (0.13)^∗∗^	0.40 (0.19)	0.38 (0.13)
	III	11	0.58 (0.04)^∗∗^	0.53 (0.30)	0.58 (0.14)
	II	15	0.49 (0.16)	0.58 (0.14)	0.40 (0.16)
	V	15	0.60 (0.09)^∗∗^	0.58 (0.14)	0.38 (0.09)
	VI	16	0.55 (0.11)	0.60 (0.19)	0.45 (0.11)
C12	I	14	0.53 (0.05)	0.48 (0.16)	0.48 (0.14)
	IV	20	0.89 (0.06)^∗∗^	0.63 (0.15)	0.13 (0.09)^∗∗^
	III	10	0.55 (0.10)	0.53 (0.24)	0.45 (0.14)
	II	15	0.51 (0.06)	0.43 (0.14)	0.33 (0.19)^∗^
	VI	15	0.55 (0.07)	0.53 (0.19)	0.50 (0.20)
	V	15	0.58 (0.04)^∗∗^	0.43 (0.26)	0.38 (0.25)
H21	I	12	0.48 (0.09)	0.58 (0.21)	0.58 (0.11)
	II	14	0.49 (0.15)	0.50 (0.13)	0.50 (0.09)
	III	10	0.48 (0.11)	0.60 (0.16)	0.38 (0.23)
	IV	20	0.49 (0.10)	0.38 (0.25)	0.38 (0.22)
	VI	15	0.47 (0.08)	0.45 (0.19)	0.75 (0.13)^∗∗^
	V	17	0.49 (0.07)	0.48 (0.10)	0.90 (0.16)^∗∗^


*The mean percentages were calculated using data from the last five sessions of each condition. Standard deviations are shown in parentheses. Note that the percentage which reached significant level was different between probe A and probes B and C due to the different number of presentation (see **Table 1** for further detail of the number of presentation of probe A in each condition). ^∗^*p* < 0.05, ^∗∗^*p* < 0.01.*

There were at least two limitations to Experiment 1. One limitation is that the probability of reinforcement was 100% in probe A but was 50% in the others. Furthermore, the number of probe A was four or eight times more than the other probes in order to maintain weight levels without post-session feeding. The other limitation was that the effects of the experiences in the previous condition might be carried over to the subsequent conditions. This could make the effects of the behavioral experiences ambiguous. In Experiment 2, we addressed these problems by alternately conducting the baseline condition and other experimental conditions, and by equalizing the reinforcement probability and the number of probe A with other probes.

## Experiment 2

### Methods

#### Subjects and Apparatus

Four pigeons (*Columba livia*), numbered B13, B21, D12, and D34, were maintained at about 80% of their free-feeding weights. Each had previous experience with various experimental procedures. The apparatus was the same as in Experiment 1.

### Procedure

#### Preliminary Training

The subjects were trained to key pecking response on a FR schedule. The response requirement was gradually increased from 1 to 20 across sessions. Session continued until 60 reinforcers were delivered and were conducted 6 days a week at approximately the same time each day. Mean completion time of FR 20 within the 20th and 80th percentile was adopted as the duration of trial C and the initial link of probe C in main training. The duration was 9.4-s in B13, 4.5-s in B21, 7.2-s in D12, and 5.6-s in D34. After pre-training, pigeons were exposed to the adjusting procedure to determine the value of the higher-cost key in the same manner as Experiment 1, except that the higher-cost value was not reset at the start of each session (i.e., the higher-cost value of the last block of the previous day was assigned to the initial higher-cost value). The value was determined as FR 6 in B13, B21, and D34, and FR 5 in D12, respectively.

#### Main Training

All sessions ended after 88 trials in all conditions (see **Table 1**). Each trial was separated by 15-s ITI. The main differences from Experiment 1 were twofold. First, both the probability of reinforcement and the number of probe A were equalized with those of probes B and C. Second, condition I and the other conditions were alternately conducted to minimize the carry-over effect from the previous condition. The order of the condition was counterbalanced across all pigeons.

#### Condition I (Baseline Condition)

Condition I consisted of trial C and three types of probe. The purpose of condition I was the same as Experiment 1, which was “to test whether the value of the higher-cost was appropriate and whether pigeons committed the sunk cost fallacy without any behavioral experiences.” All pigeons were exposed to condition I at first and again after each of the other experimental conditions. If the percentage choice of the higher-cost key in probe A was more than 10% in the first exposure, the value was gradually increased until it was stable at less than 10%. In the subsequent exposure, condition I ended when the percentage choice of the higher-cost key in probe A was stable at less than 10%.

#### Condition II

Condition II consisted of trials B and C, and probes. Trial B was the same as that in Experiment 1, and it was expected that the lower-cost key would be devalued because all responses to the lower-cost color had been extinguished.

#### Condition III

Condition III consisted of trials A3 and C, and three probes. Since trial A1 did not have the effect of increasing the higher-cost preference in Experiment 1, trial A1 was developed into trial A3. In trial A3, the higher-cost color was presented on the center key and after FR 20 + *X* responses, a reinforcer was delivered at 100% for 3-s. The value *X* was the same value as the higher-cost key for each subject. For example, in the case of B13, the higher-cost value was 6 such that the reinforcer was presented after 26 (20 + 6) responses.

#### Condition IV

Condition IV consisted of trials A3 and B, and probes. This condition therefore created both experiences related to the lower- or higher-cost key.

Each condition lasted for a minimum of 10 sessions and until no systematic trend in preference was observed in each probe for at least three consecutive sessions. In addition, condition I required that the mean percentage choice of the higher-cost key over the last five sessions was stable at less than 10% in probe A.

### Results and Discussion

**FIGURE 3:**
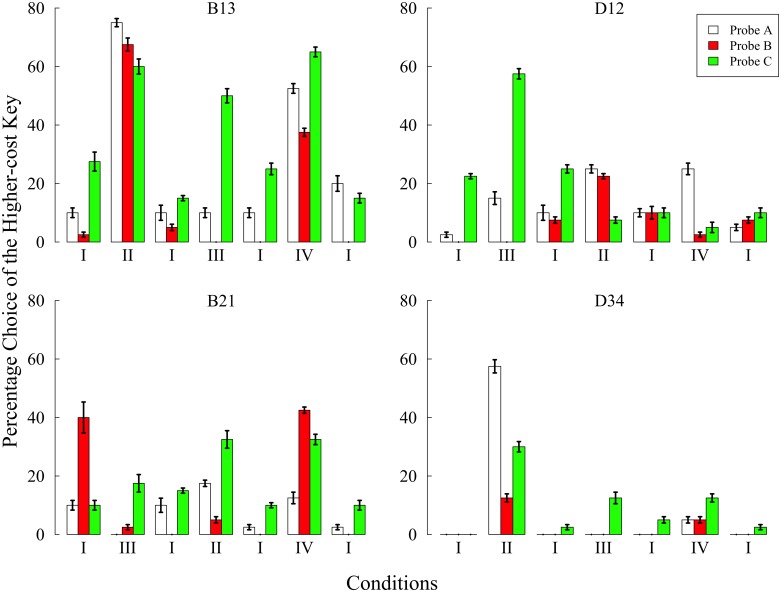
**Preference for the higher-cost key in each probe over the last five sessions of each condition in Experiment 2.** Choice percentages were calculated in each probe by dividing the number of choices of the higher-cost key by the total number of choices. Error bars represent standard errors.

**Figure 3** shows the preference for the higher-cost key in each condition. Compared with the results of Experiment 1, it is notable that higher-cost preference was more clearly increased when pigeons had certain behavioral experiences. The main difference from Experiment 1 was that the probability of reinforcement and the number for probe A were equalized with the other probes. Therefore, it is apparent that this manipulation made the effects of the behavioral experiences on preference clear.

The most remarkable increase in the higher-cost preference was observed in conditions II and IV, both of which included trial B. In condition II, three pigeons other than D12 showed increased preference in all probes compared with the baseline condition. However, the strongest preference was shown in either probe A or C in all pigeons. In condition IV, three pigeons other than D34 also showed a higher-cost preference in one or more probes. It is notable that only B21 showed the highest preference for the higher-cost key in probe B. However, B21 chose the right key in probe B almost 100% of the time (see **Table 3**), and this position preference was closely related to the preference for the higher-cost key. One might consider that B21’s performance in condition IV was caused by this position preference but not by the behavioral experiences created in condition IV so that this result could not be an evidence of the sunk cost fallacy. The similar position preference was also observed in the other subjects, and most of them were closely related to the higher-cost preference. However, the position preference was not observed in the baseline condition conducted before and after condition IV. As noted below, an exception was the last baseline condition of B13. Except for this case, these facts suggest that the results from B21 in condition IV were also caused by behavioral experiences, and therefore could be evidence of the sunk cost fallacy.

**Table 3:** The order of the condition, the number of sessions, and the mean percentage choice of the left key in each probe of each condition in Experiment 2.

Subjects	Conditions	Number of sessions	Percentage choice of the left key		
			
			Probe A	Probe B	Probe C
B13	I	10	0.65 (0.16)	0.38 (0.20)	0.53 (0.24)
	II	15	0.98 (0.06)^∗∗^	0.15 (0.10)^∗∗^	0.70 (0.23)^∗^
	I	38	0.50 (0.27)	0.58 (0.23)	0.48 (0.24)
	III	18	0.68 (0.14)^∗^	0.58 (0.11)	0.63 (0.23)
	I	32	0.68 (0.19)^∗^	0.30 (0.07)^∗^	0.68 (0.14)^∗^
	IV	32	0.75 (0.43)^∗∗^	0.78 (0.44)^∗∗^	0.50 (0.32)
	I	100	0.75 (0.18)^∗∗^	0.50 (0.13)	0.48 (0.10)
B21	I	10	0.40 (0.16)	0.30 (0.29)^∗^	0.43 (0.26)
	III	10	0.45 (0.11)	0.55 (0.14)	0.55 (0.17)
	I	45	0.38 (0.18)	0.48 (0.16)	0.55 (0.19)
	II	12	0.38 (0.09)	0.35 (0.16)	0.65 (0.24)
	I	23	0.48 (0.21)	0.50 (0.23)	0.58 (0.23)
	IV	17	0.55 (0.14)	0.03 (0.06)^∗∗^	0.48 (0.14)
	I	36	0.38 (0.20)	0.43 (0.07)	0.53 (0.19)
D12	I	10	0.38 (0.23)	0.50 (0.15)	0.60 (0.21)
	III	10	0.50 (0.13)	0.63 (0.20)	0.75 (0.00)^∗∗^
	I	23	0.38 (0.23)	0.58 (0.19)	0.65 (0.24)
	II	17	0.48 (0.10)	0.78 (0.10)^∗∗^	0.48 (0.16)
	I	19	0.40 (0.10)	0.60 (0.16)	0.48 (0.16)
	IV	14	0.53 (0.27)	0.45 (0.19)	0.48 (0.27)
	I	24	0.55 (0.07)	0.45 (0.19)	0.48 (0.10)
D34	I	10	0.65 (0.16)	0.60 (0.10)	0.53 (0.10)
	II	15	0.43 (0.38)	0.50 (0.09)	0.25 (0.15)^∗∗^
	I	24	0.58 (0.14)	0.48 (0.10)	0.48 (0.10)
	III	10	0.48 (0.16)	0.58 (0.07)	0.33 (0.19)^∗^
	I	10	0.63 (0.15)	0.50 (0.09)	0.50 (0.18)
	IV	10	0.33 (0.24)^∗^	0.35 (0.14)	0.38 (0.28)
	I	10	0.45 (0.14)	0.50 (0.20)	0.53 (0.22)


*The mean percentages were calculated using data from the last five sessions of each condition. Standard deviations are shown in parentheses. ^∗^*p* < 0.05, ^∗∗^*p* < 0.01.*

The higher-cost preference was also observed in condition III, in which responses to the higher-cost key was reinforced at a rate of 100% for 3-s after FR 20 + *X* responses. All pigeons showed a remarkable increase in the preference in probe C, but no subject chose the higher-cost key in probe B. Thus, the experiences related to the higher-cost key again failed to cause the sunk cost fallacy.

In Experiment 2, the baseline condition was conducted before and after other experimental conditions. Except for B13’s last exposure, the higher-cost preference decreased to less than 10% in probe A. Since B13 consistently preferred the left side key, the percentage choice in probe A did not decrease to less than 10% even after 100 sessions (see **Table 3**). It is also noteworthy that B21 preferred the higher-cost key in probe B of the first baseline condition. Although this result may appear to provide evidence of the sunk cost fallacy, there was no such tendency in the subsequent baseline condition. It is therefore more plausible to explain this tendency by appealing to the instability of the choice behavior. Thus, as demonstrated in Experiment 1, the sunk cost fallacy did not occur when subjects did not have any behavioral experiences related to the lower- and/or higher-cost key.

Note that some pigeons had continued to choose the higher-cost alternative in probe C of the second baseline condition. Visual observation during experimental sessions revealed that all pigeons had pecked either key during the initial link of probe C and trial C. This tendency might contribute to the higher-cost preference in probe C. In some cases, for example, subject D34 started to peck the left side key during the initial link and continued to peck even after the terminal link started.

**FIGURE 4:**
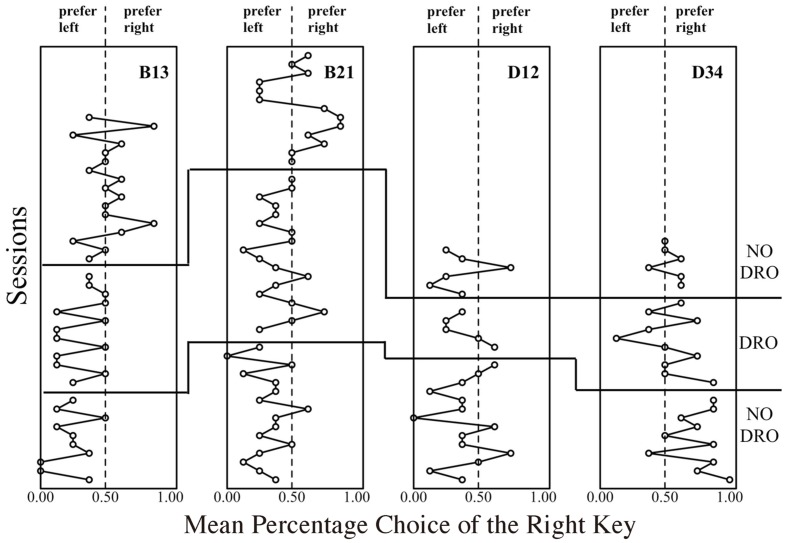
**Mean percentage choice of the right side key in probe C of the second baseline condition.** The plots on the left side of the dashed line show the extent to which subject preferred the left side key, and vice versa.

Given that the lower- and higher-cost colors were quasi-randomly assigned to either the left or the right key for every probe trial, the higher-cost preference should be stable around 0.5. In fact, the higher-cost preference of D34 was stable around 0.5 and did not decrease even after 10 sessions. To extinguish this tendency, a certain manipulation was applied to the response to either the left or side key. Under this manipulation, the terminal link never started as long as the pigeon continued to peck either the left or right side key (technically, a differential-reinforcement-of-other-behavior, DRO, schedule). Therefore, this was expected to reduce the superstitious tendency. **Figure 4** shows the change of the percentage choice of the one side key before and after the DRO introduction. Two pigeons, B13 and B21, tended to choose the left key; one pigeon, D34, choose the right key until the first 10–15 sessions. During DRO schedule application, however, the peck to the dark side key gradually ceased and one side key preference also decreased. Even after the removal of DRO schedule, this tendency did not recur, and the mean percentage choice of the higher-cost key was stable at a low level. This suggests that one cause of the preference in probe C resulted from the superstitious behavior during the initial link.

In sum, the results from Experiment 2 suggest that previous behavioral experiences may cause the sunk cost fallacy. However, the sunk cost fallacy was observed only in B21, and it is still unclear what experiences, from trial A3, trial B, or through their interaction, resulted in the sunk cost fallacy. In Experiment 3, we addressed these problems and also examined the effect of the initial responses (i.e., the sunk cost) on choice behavior in probe B. Across Experiments 1 and 2, the initial responses in probe B was fixed at FR 20. Some studies suggested that the amount of the initial responses influenced performance in the choice situation (e.g., Garland, 1990; Garland and Newport, 1991; Pattison et al., 2012; Magalhães and White, 2014). It is possible that the initial 20 responses were not appropriate for showing the effect of the behavioral experiences in probe B. We therefore changed the initial investment in probe B from FR 20 to FR 10 in Experiment 3.

## Experiment 3

### Methods

#### Subjects and Apparatus

Four pigeons (*Columba livia*), numbered B31, C21, D23, and H14 were maintained at about 80% of their free-feeding weights. Each had previous experience with various experimental procedures. The apparatus was the same as in Experiments 1 and 2.

### Procedure

#### Preliminary Training

The subjects were trained to key pecking response on a FR schedule. The response requirement was gradually increased from 1 to 10 across sessions. Sessions continued until 60 reinforcers were delivered and were conducted 6 days a week at approximately the same time each day. Mean completion time of FR 10 within the 20th and 80th percentile was adopted as the duration of trial C and the initial link of probe C. The durations were 4.9, 7.2, 8.5, and 4.2-s in B31, C21, D23, and H14 respectively. After pre-training, the higher-cost value was determined for each pigeon in the same manner as in Experiment 2. The value was FR 5 in B31, C21, and D23, and FR 6 in H14.

#### Main Training

Each session ended after 88 trials in all conditions (see **Table 1**) and trials were separated by 15-s ITI. Each condition lasted for a minimum of 10 sessions and until the following stability criteria were met. First, no systematic trend in preference was observed in all probes for at least three consecutive sessions. Second, the range of mean percentage choice of the higher-cost key for each probe was less than 25% during the last five sessions. In addition, only condition I required that the percentage choice of the higher-cost key should be stable at less than 10% in all probes. This requirement was adopted to more clearly compare the pigeons’ performance across conditions. All subjects were first exposed to condition I; thereafter, they were alternately exposed to the three experimental conditions and the baseline condition. The order of the condition was counterbalanced across pigeons.

#### Condition I (Baseline Condition)

Condition I was the same as in Experiment 2. This condition consisted of trial C and three types of probes and did not create any behavioral experiences to the lower- and higher-cost key. All pigeons were exposed to condition I at first and again after each of the other experimental conditions.

#### Condition II

Condition II consisted of trials B and C, and three probes. This condition was the same as condition II of Experiment 2 so that pigeons experienced responses to the lower-cost color resulted in no food.

#### Condition III

Condition III consisted of trials A4 and C, and three probes. Since trial A3 failed to increase the higher-cost preference in Experiment 2, we developed trial A3 into trial A4. The notable difference was that the reinforcement duration was changed from 3 to 6-s. Along with the change of probe B, required responses were also changed from FR 20 + *X* to FR 10 + *X*. Thus, trial A4 created the experience that responses to the color related to the higher-cost key resulted in a longer time for food presentation.

#### Condition IV

Condition IV consisted of trials A4 and B, and three probes. Therefore this condition was identical to condition IV of Experiment 2, except that trial A3 was changed to trial A4.

### Results and Discussion

**FIGURE 5:**
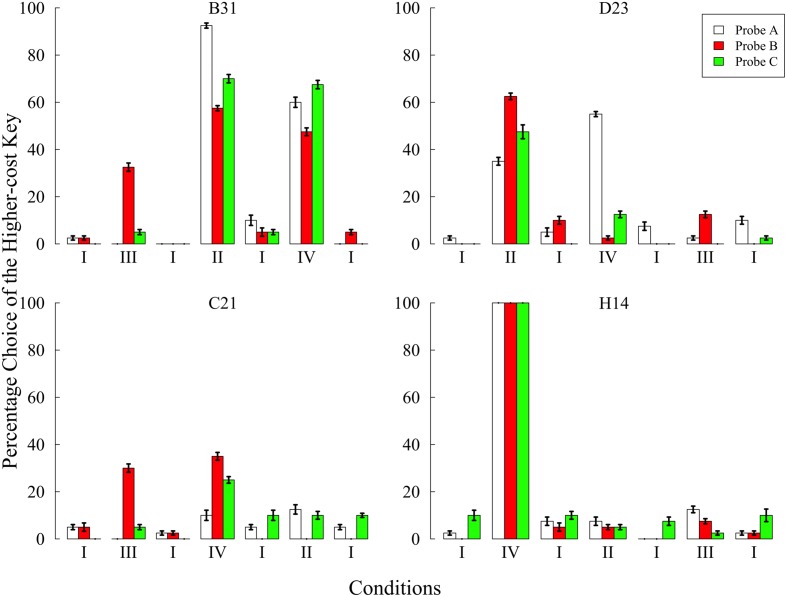
**Preference for the higher-cost key in each probe over the last five sessions of each condition in Experiment 3.** Choice percentages were calculated in each probe by dividing the number of choices of the higher-cost key by the total number of choices. Error bars represent standard errors.

**Figure 5** shows the preference for the higher-cost key in each condition. The preference increased in one or more conditions without the baseline. In particular, the results from condition III clearly show the occurrence of the sunk cost fallacy.

In condition III, three of the four pigeons preferred the higher-cost key in probe B but did not prefer it in probe A and C. Although, C21 strongly preferred the left side key in probe B (see **Table 4**), this subject did not show preference in the other probes. In addition, the position preference, as in Experiment 2, ceased in the baseline condition conducted after condition III. These results seem to suggest that position preference also resulted from the behavioral experience created in condition III, despite the fact that the position preference resulted in an increased percentage of higher-cost key choice. These facts may support the conclusion that C21 committed the sunk cost fallacy.

**Table 4:** The order of the condition, the number of sessions, and the mean percentage choice of the left key in each probe of each condition in Experiment 3.

Subjects	Conditions	Number of sessions	Percentage choice of the left key		
			
			Probe A	Probe B	Probe C
B31	I	12	0.55 (0.11)	0.55 (0.23)	0.58 (0.33)
	III	10	0.50 (0.15)	0.68 (0.14)^∗^	0.38 (0.25)
	I	11	0.40 (0.19)	0.53 (0.16)	0.38 (0.09)
	II	28	0.53 (0.14)	1.00 (0.00)^∗∗^	0.55 (0.14)
	I	17	0.50 (0.27)	0.48 (0.14)	0.53 (0.06)
	IV	51	0.98 (0.06)^∗∗^	1.00 (0.00)^∗∗^	1.00 (0.00)^∗∗^
	I	36	0.45 (0.24)	0.55 (0.19)	0.53 (0.16)
C21	I	10	0.63 (0.15)	0.48 (0.06)	0.48 (0.16)
	III	11	0.38 (0.18)	0.83 (0.21)^∗∗^	0.55 (0.14)
	I	10	0.48 (0.21)	0.65 (0.24)	0.50 (0.20)
	IV	35	0.70 (0.33)	0.85 (0.10)^∗∗^	0.58 (0.21)
	I	10	0.48 (0.30)	0.48 (0.16)	0.35 (0.16)
	II	12	0.60 (0.06)	0.48 (0.14)	0.60 (0.24)
	I	12	0.63 (0.20)	0.55 (0.21)	0.58 (0.21)
D23	I	10	0.50 (0.15)	0.48 (0.06)	0.48 (0.10)
	II	20	0.08 (0.07)^∗∗^	0.13 (0.15)^∗∗^	0.00 (0.00)^∗∗^
	I	21	0.53 (0.14)	0.50 (0.18)	0.53 (0.14)
	IV	24	0.03 (0.06)^∗∗^	0.45 (0.23)	0.43 (0.23)
	I	36	0.38 (0.13)	0.50 (0.25)	0.55 (0.07)
	III	11	0.43 (0.26)	0.55 (0.14)	0.53 (0.16)
	I	12	0.45 (0.11)	0.53 (0.16)	0.50 (0.18)
H14	I	31	0.43 (0.14)	0.53 (0.21)	0.53 (0.10)
	IV	10	0.60 (0.10)	0.53 (0.24)	0.58 (0.26)
	I	43	0.43 (0.07)	0.38 (0.18)	0.35 (0.16)
	II	23	0.53 (0.10)	0.63 (0.32)	0.33 (0.29)^∗^
	I	10	0.50 (0.20)	0.43 (0.19)	0.48 (0.24)
	III	19	0.40 (0.16)	0.45 (0.24)	0.40 (0.10)
	I	11	0.50 (0.00)	0.53 (0.06)	0.38 (0.20)


*The mean percentages were calculated using data from the last five sessions of each condition. Standard deviations are shown in parentheses. ^∗^*p* < 0.05, ^∗∗^*p* < 0.01.*

In condition IV, although all subjects preferred the higher-cost key regardless of the type of probes, there was no consistent trend across subjects. The patterns of preference in B31 and C21 were similar to that of conditions II and III, respectively, suggesting that C21 again committed the sunk cost fallacy. The difference between those pigeons might reflect the fact that B31 was more affected by trial B, while C21 was more affected by trial A4.

Of greater interest was the finding that H14 exclusively chose the higher-cost key in all probes. This exclusive preference emerged from the fifth session and continued until the 10th session of condition IV, but was gradually decreased after returning to the baseline condition. After that, H14 never preferred the higher-cost key. It was unclear whether H14 committed the sunk cost fallacy because of the ceiling effect. It was only evident that the behavioral experiences in condition IV strongly affected H14’s preference.

The results of condition II were similar to those of Experiment 2. Pigeons except for H14 increased their preference for the higher-cost key in this condition. It is worth noting that D23 showed the strongest preference in probe B. However, the fact that D23 exclusively chose the left side key in all probes makes it difficult to conclude that D23 committed the sunk cost fallacy. The other pigeons showed an overall increase in the higher-cost preference across all probes.

In summary, three of the four pigeons committed the sunk cost fallacy in condition III and only C21 committed the fallacy in conditions III and IV. These findings indicate that the behavioral experience in trial A4 resulted in committing the sunk cost fallacy. The results of condition II showed that the experiences from trial B increased the preference across all probes for B31 and D23. This was consistent with the results of Experiments 1 and 2.

## General Discussion

We strictly defined the sunk cost fallacy based on past studies (e.g., Arkes and Blumer, 1985; Arkes and Ayton, 1999) and then created an experimental analog for pigeons according to that definition. In the section “Introduction,” we pointed out that the Navarro and Fantino’s (2005) procedure did not satisfy the third requirement and the importance of distinction between indiscriminability and irrationality. In the present study, at least following two conditions demonstrated that pigeons “knew” the irrational alternative. First, both the lower- and higher-cost alternatives were simultaneously presented in each probe trial. This was the prerequisite for discriminating the better alternative. Second, pigeons did not show the higher-cost preference in the baseline condition. These conditions clearly demonstrated that the experimental procedure used in the present study met the third requirement.

It might be argued that probe B used in the present study did not satisfy the first requirement, “continuing the investment because of the past investment (i.e., the sunk cost),” because choosing the higher-cost key in the terminal link should not be considered as “continuing” investment in the higher-cost one. The reasons for this interpretation are as follows: Frist, the location of the higher-cost alternative differed while the color was the same between the initial and terminal links. However, in condition 3 of Experiment 3, three of four pigeons showed the higher-cost preference only in probe B but not in probes A and C, clearly suggesting that pigeon’s choice in the terminal link was affected by the color of the higher-cost key. Although this may be an *ex post facto* interpretation, some studies also have been considered choosing the alternative with different location from the initial link as continuing investment (e.g., Pattison et al., 2012, Experiment 1). Thus, it seems reasonable to consider the choice of the same color with different location from initial link as “continuing” investment and to consider that probe B satisfied the first requirement. Another reason is related to the relationship between the investment in the initial and terminal links. In probe B, the number of responses emitted in the initial link did not affect the required number of responses in the terminal link. This implies that the additional responses to the higher-cost key in the terminal link seem to be independent of the initial investment. However, note that we did not manipulate the amount of the initial investment across trials or conditions in each experiment of the present study (i.e., FR 10 in Experiment 3 and FR 20 in Experiments 1 and 2). If the amount of the initial investment differs across trials or conditions, the required number of responses in the terminal link should be varied depending on the emitted responses in the initial link. In other words, if the amount of the initial investment is constant across trials or conditions, the additional investment in the terminal link should also be constant. Thus, it cannot be evidence of independency between the initial and terminal investments (see also discussion about the effect of the amount of initial investment on the choice below).

The following general conclusions can be drawn concerning the relationship between behavioral experiences and the sunk cost fallacy. Three of four pigeons committed the sunk cost fallacy in condition III of Experiment 3, in which they experienced trial A4, which created the experience that responses to the higher-cost key produced a longer time for food presentation. Although trial A1 of Experiment 1 and A3 of Experiment 2 created similar experiences, the reinforcement duration was 3-s in both trials. In addition, trial A1 required additional responses after reinforcement to terminate the current trial. These factors might have prevented the sunk cost fallacy in Experiments 1 and 2.

Pigeons also increased the higher-cost preference in the experimental conditions including trial B. Across all experiments, trial B created the same experience, namely that responding to the lower-cost key produced no food. However, this experience increased the preference in all probes for some pigeons, and not only in probe B. We therefore concluded that the experiences created by trial B increased the overall preference for the higher-cost key, but did not cause the sunk cost fallacy themselves. On the other hand, pigeons did not show a preference for the higher-cost key in the baseline condition, in which they did not have any behavioral experiences related to either the lower- or higher-cost key.

Taken together, these results show that the experiences relating to the higher-cost alternative was the cause of the sunk cost fallacy being committed in our experiments. However, we should consider another possibility before concluding that the experience from trial A4 was the only cause of the sunk cost fallacy. In Experiment 3, the initial responses of probe B (sunk cost) were also changed from FR 20 to FR 10. Thus, it is possible that a small number of initial responses relative to Experiments 1 and 2 also had effects on choice behavior in probe B.

In animal studies, Pattison et al. (2012) examined the relationship between the amounts of initial investment and persistence and reported that pigeons were more likely to persist in one alternative as the required responses in the initial link (i.e., sunk cost) increased. However, as Magalhães and White (2014) pointed out, the required responses in the choice situation varied depending on the responses in the initial link, such that the choice could be influenced by both the initial responses and the decreased requirement in the choice situation. Magalhães and White (2014) also examined this question using a different procedure from Pattison et al. (2012). In their Experiment 3, they manipulated the initial investment and found that the higher investments enhanced the sunk cost fallacy. However, the difference of the initial investment values between two conditions was more drastic (FR 1 versus FR 35) than that of our study (FR 10 versus FR 20). This difference might contribute to the difference in the results. The question of how much initial responses influence the subjects’ choice should be examined more precisely in future research.

Some limitations require consideration concerning this study. Although we carefully manipulated some variables and examined the cause of higher-cost preference across experiments, the present study is still preliminary for the following reasons. We defined the rational choice as that the choice percentage of higher-cost key was stable at less than 10% and paid attention to whether the choice percentage increased and stable at more than 10% in each experimental condition. However, this standard was subjective and lacked the objective ground. Furthermore, three out of four pigeons showed more than 10% preference in condition III of Experiment 3, but the percentage did not reach 50%. It is obvious that the sunk cost fallacy observed in the present study was relatively weak compared to previous studies (e.g., Arkes and Blumer, 1985).

It also is difficult to explain the large individual differences in preference and the position preference shown in probes. Of importance was the position preference observed across all experiments. This provides one possible explanation for the results: the preference for the higher-cost key resulted from position preferences and not from behavioral experiences. However, there is some evidence against this explanation. Across experiments, the lower- and higher-cost colors in all probes were quasi-randomly assigned to either side key for every probe. This means that if pigeons consistently choose one side key, they must choose the higher-cost key in almost half of all probes. In other words, if pigeons did prefer the lower-cost key, they must not show such a position preference. In addition to this, the position preference occurred only when pigeons had behavioral experiences related to either the lower- or higher-cost key. Except for the result of B13 from Experiment 2, position preference ceased when returning to the baseline condition in Experiments 2 and 3. These facts suggest that, even though the preference for the higher-cost key resulted from position preference, it was also induced by particular behavioral experiences.

There was also another problem that the results of the present experiments might be strongly affected by the order of experimental conditions. The sunk cost fallacy observed in Experiment 3 was robust in two subjects, B31 and C21, more than in D23. The order of the experimental conditions had some effect on these results: B31 and C21 were exposed to condition III after the first baseline condition, while D23 was exposed only after the third baseline condition. One possible reason for this is that the many occasions of probes through the sessions made the higher-cost preference harder to increase. In our experiments, each probe was presented in each session so that the experienced number of probes also increased as experiment progressed. The results of Experiment 1 support this explanation. Experiment 1 had many occasions of probe A, and Experiment 2 revealed that this was one of the causes that the higher-cost preference was unlikely to increase across experimental conditions.

It also should be noted some differences between procedure used in the present study and used in previous study for human participants: the most important difference was the consequence of choice. In human cases, participants are required to choose whether continuing or quitting the investment and no consequences occur if they choose quitting the investment. For example, in the case of airplane construction, the airplane is not realized if quitting the investment is chosen. On the other hand, in the present study, pigeons were required to choose one of lower- or higher-cost alternatives and could get the same amount of food according to the same probability of reinforcements by quitting the investment (i.e., choosing the FR 2 schedule) as in the case that they chose to continuing the investment (i.e., choosing the FR *X* schedule). Therefore, it is necessary to further examine whether human participants also commit the sunk cost fallacy in the choice situations similar to the present study.

Despite these limitations, the present study suggests the possibility that pigeons also commit the sunk cost fallacy in situations that are highly similar to human cases. It also implies the possibility that behavioral experiences might be one of the common causes of the sunk fallacy across human and non-human animals. Note that more than one cause might relate to the sunk cost fallacy, and some causes might be effective only in humans. Arkes and Ayton (1999), for example, pointed out that the overgeneralization of rules such as “don’t waste” is responsible in humans. Nevertheless, it is obviously important to examine the common causes across humans and animals for understanding the mechanism of the sunk cost fallacy. The strict definition and the experimental analog developed in the present study would be helpful in examining the common cause across species.

## Author Contributions

SF and TS conceived the design of experiment. SF conducted the experiments, analyzed the data, and wrote the first draft of the manuscript. SF and TS contributed equally to later drafts and approved the final manuscript.

## Conflict of Interest Statement

The authors declare that the research was conducted in the absence of any commercial or financial relationships that could be construed as a potential conflict of interest.
